# ECS: Efficient Communication Scheduling for Underwater Sensor Networks

**DOI:** 10.3390/s110302920

**Published:** 2011-03-04

**Authors:** Lu Hong, Feng Hong, Zhongwen Guo, Zhengbao Li

**Affiliations:** Department of Computer Science, Ocean University of China, Qingdao 266100, China; E-Mails: honglu@ouc.edu.cn (L.H.); guozhw@ouc.edu.cn (Z.G.); lizhengbao@ouc.edu.cn (Z.L.)

**Keywords:** underwater sensor networks, communication scheduling, TDMA, ST-MAC

## Abstract

TDMA protocols have attracted a lot of attention for underwater acoustic sensor networks (UWSNs), because of the unique characteristics of acoustic signal propagation such as great energy consumption in transmission, long propagation delay and long communication range. Previous TDMA protocols all allocated transmission time to nodes based on discrete time slots. This paper proposes an efficient continuous time scheduling TDMA protocol (ECS) for UWSNs, including the continuous time based and sender oriented conflict analysis model, the transmission moment allocation algorithm and the distributed topology maintenance algorithm. Simulation results confirm that ECS improves network throughput by 20% on average, compared to existing MAC protocols.

## 1. Introduction

Underwater acoustic sensor networks (UWSNs) have a promising future in the area of information collection with more and more applications in recent years, such as ocean environmental surveillance, resource exploration and disaster prevention [1–3]. Unlike terrestrial sensor nodes that rely on radio waves to communicate with each other, underwater sensor nodes utilize acoustic waves to transmit data, which constitutes a significant difference between underwater sensor networks and terrestrial sensor networks (TSNs).

First, underwater sensor nodes consume much more energy in transmission than terrestrial sensor nodes, not only that used in reception, but also in transmission [3]. This phenomenon makes the confliction of packets more unacceptable in UWSNs than in TSNs. Second, the transmission range of an acoustic modem (2–4 km) is much greater than that of an RF modem (150 m). This feature leads to that circumstance whereby the transmitter cannot detect the confliction at the receiver. Meanwhile, the propagation speed of acoustic signals in underwater environments is about 1,500 m/s, which is thousands of times slower than RF propagation (*i.e.*, 3 × 10^8^ m/s) [4,5]. The channel status in short-range RF networks can only be measured by the transmission time; however, the propagation delay cannot be ignored in UWSNs.

The unique characteristics of UWSNs bring about new challenges for MAC protocol design. Great energy consumption in transmission produces collision free protocols more suitable for UWSNs. Long communication ranges make the receiver the only point where the packets’ confliction is detected. However, the long propagation delay makes handshake protocols like RTS and CTS inefficient for UWSNs, as they would greatly decrease the network throughput.

Consequently, TDMA protocols are of great importance in UWSNs. Many TDMA approaches have been proposed for UWSNs, largely falling into two categories: one-slot approaches and multi-slot approaches, depending on how many time slots can be exploited to finish transmission of one packet between one-hop neighbors. One-slot approaches require the transmission to be accomplished in a single slot, so the length of a time slot should be designed to be at least one frame time plus the longest propagation delay of all links in the transmission range [6,7]. Idle time will happen in one slot when the transmission is between two neighbors at a short distance, which will eliminate the throughput. Multi-slot TDMA approaches use more than one slot to accomplish the transmission of one packet between two neighbors. ST-MAC [8] assumes that the propagation delay of any link must be integral to one slot time, which is set to the time to transmit one frame. Under this assumption, ST-MAC exploits a centralized heuristic algorithm to allocate time slots for multi-hop networks and achieves better efficiency.

The core of TDMA protocols is to assign different transmission moments to transmitters. However, previous approaches are all based on allocating discrete time slots to nodes. If we treat the allocation problem of transmitting moment to nodes as one continuous function, we can further eliminate the idle time between the transmissions of different pairs of neighbors, and improve the throughput of the whole system, which is the motivation of this paper. Continuous allocation of transmitting moments brings new challenges to the MAC protocol design: the local conflict graphs (LCG) of the nodes have to be constructed based on continuous time and the transmission forbidden time of each node should be calculated as a continuous period. After that, continuous transmitting moments have been allocated to each node according to the transmission forbidden time of these nodes, which is an NP-hard problem to ascertain the optimum global allocating scheme.

In this paper, we propose the Efficient Continuous Scheduling algorithm (ECS) to solve the MAC problem of UWSNs. ECS contains three parts: a transmitting forbidden time calculation algorithm based on nodes’ local conflict graph (LCG), an allocation algorithm for nodes to decide their own transmitting moment based on a group of heuristic rules, and a distributed maintenance problem to solve the situations of neighbor death or joining of new nodes. Major contributions of this paper are:

1. Aimed at the unique phenomenon of UWSN MAC issue that the delay differences of dissimilar links could not be ignored, a sender oriented conflict model based on continuous time allocation is proposed. A distributed algorithm to generate local conflict model (LCG) is also advanced based on this model.
2. An efficient TDMA protocol (ECS) for UWSNs based on a group of heuristic rules is proposed. According as LCG, a node uses degree, load and link delay to calculate priority, and selects its transmission moment in priority order. The ECS algorithm could reduce the running time of nodes’ transmission moment allocation.
3. Compared to slotted schemes, continuous time based allocation could reduce idle times of receivers and improve network throughput. Simulation results confirm that ECS improves network throughput by 20% on average compared to existing MAC protocols.

The rest of the paper is organized as follows. Section 2 discusses the related work. We present ECS in detail in Section 3. Section 4 shows the simulation results and we conclude our work in Section 5.

## 2. Related Work

As the transmission costs great energy in UWSNs, all existing MAC approaches try to avoid packet collisions, and can be further divided into three categories: (1) contention-based MAC without RTS/CTS, (2) contention-based MAC with RTS/CTS, and (3) contention-free MAC.

**Contention-based MAC without RTS/CTS**: This kind of protocol is a modified Aloha protocol. A short tone or preamble is used as transmitting notification to neighbor nodes. When a node hears the transmitting notification of other nodes, it will back off its own transmission randomly [9] or reschedule its own transmission based on the knowledge of all its neighbors’ notification [10]. However, such a notification scheme wastes channel bandwidth and energy.

**Contention-based MAC with RTS/CTS**: this kind of protocol exploits virtual carrier sense to save energy and avoid conflicts. Nodes pick up with data information in control packets to help other nodes in calculating the “busy time” of a channel, and stop listening in this period of time [11,12]. Due to the long propagation delay in underwater environments, sensor nodes must wait for the long round-trip time of RTS/CTS exchange, and cannot send any data. In order to decrease the waiting time, these works further exploit the idle period of RTS/CTS exchange to send data packets or other RTS messages if they would not collide with RTS/CTS.

**Contention-free protocols**: Several works have shown that, due to the long propagation delay in underwater environments, it is difficult for the contention-based schemes to approximate the optimal energy-efficient MAC in UWSNs. Hence, contention free approaches such as FDMA, CDMA, or TDMA have attracted much attention. FDMA divides the frequency band into several sub-bands, however, the narrow band of the underwater acoustic channel results in a low throughput (e.g., 50 bits/s) [13]. CDMA approaches have been proposed [13–15], however, they have an inherent near-far problem which cannot be well addressed, especially for the long propagation delay and long communication range of UWSNs.

Therefore, TDMA protocols have attracted a lot of attention, falling into two categories: one-slot approaches and multi-slot approaches. One-slot approaches require that the transmission must be accomplished in a single slot, so the length of a time slot is at least one frame time plus the longest propagation delay of all links in the transmission range [6,7,14,16]. Paper [17] proposes a TDMA scheduling scheme for mobile underwater sensor nodes using an adaptive token polling, and paper [18] suggests a method that decreases energy consumption and propagation delays caused by channel collision by solving some problems of UWAN-MAC, which occur when the number of nodes increases. ST-MAC is the first multi-slot TDMA protocol especially designed for UWSNs [8], which allows more than one slot to accomplish the transmission of one packet between two neighbors. ST-MAC exploits a centralized algorithm on the spatial-temporal conflict graph (ST-CG) to assign time slots for every node. In order to keep the condition that propagation delay of each link must be integral multiple of one slot; the frame size must be designed as small as possible.

Contention based MAC approaches will cause energy wasting because of data collisions, and scheduling schemes are almost always based on time slots. They all ignore the feasibility of allocating transmission moments on a continuous time axis without slotting, which could further improve channel utilization and network throughput.

## 3. Design of ECS

In this section, we first demonstrate our metrics to design ECS in underwater environments and present the conflict model based on continuous time. Then we introduce the exact design of ECS and present basic ideas of ECS via an example. Finally we give maintenance schemes to deal with the cases of node death or joining in the network.

### 3.1. Metrics

**Figure 1.**
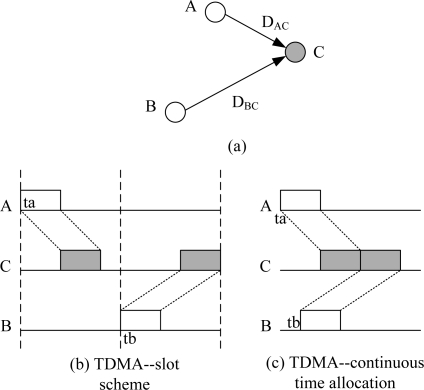
Different UWSN transmission moment allocations.

The most important problem in UWSN MAC design is the measurement of conflicts by receiving ends. See Figure 1(a) as an example, where nodes *A* and *B* want to send to *C*, and we define *D_AC_* and *D_BC_* as the propagation delay of link *AC* and *BC*, *T* is the frame time, *t_a_* and *t_b_* are the transmission moments of nodes *A* and *B*.

In TSNs, the propagation delay of an RF signal could be ignored because it is very small. We define the length of a TDMA slot as *T*, node *A* and *B* can choose different transmission slot to avoid a collision. However, in UWSNs the propagation delay is too large to be disregarded. In our example, the slot length should be at least *D_BC_* + *T* to avoid collisions. Then, for node *C*, its receiving sequence will become sparse on the time axis, as shown in Figure 1(b).

An ideal TDMA scheme is shown in Figure 1(c). If *A* chooses *t_a_* to transmit, the packet will arrive at *C* at *t_a_* + *D_AC_*. In order to minimize the idle time of node *C*, packet of node *B* should arrive at *t_a_* + *D_AC_* + *T*. So, *B*’s transmission moment *t_b_* should be *t_b_* = *t_a_* + *D_AC_* + *T* − *D_BC_*.

In conclusion, compared with traditional slot schemes, a continuous time allocation based TDMA scheme could improve network throughput and decrease idle time of receiving ends. This scheme fully utilized the characteristics of long propagation delay and short packets of UWSNs, and this are the key metrics in the design of ECS.

### 3.2. Continuous Time Based Conflict Model

We make three assumptions before discussion:

1. The nodes’ communication model is a disk model; a node’s communication range and interference range are both cyclical regions. All nodes in the network are homogenous, and they all have the same transmission radius (*TR*) and interference radius (*IR*).
2. Clock synchronization is needed for TDMA transmission scheduling. Recent works [19,20] have provided synchronization mechanisms for UWSN. Hence, the clock synchronization issue is considered out the scope of our discussion.
3. Each node could get its geographical positions from positioning devices such as GPS or use an localization algorithm to calculate it. Lots of recent research has been done on UWSN nodes’ localization such as [21] and [22], so we do not discuss localization issues in this paper.

The continuous time based conflict model is composed of local conflict graph (LCG) and the algorithm to calculate forbidden time. Now we give the related definitions:

**Definition 1: Coverage area *Cov*(*N, R*)**. A circular region in which the centre is node *N* and the radius is *R*. For example, for node *N(x_n_, y_n_)*, its communication range is:

$$\mathrm{Cov}(N,\mathrm{TR})=\left\{\left(x,y\right)|{\left(x-x_{n}\right)}^{2}+{\left(y-y_{n}\right)}^{2}⩽{TR}^{2}\right\}$$

and its interference range is:

$$\mathrm{Cov}(N,\mathrm{IR})=\left\{\left(x,y\right)|{\left(x-x_{n}\right)}^{2}+{\left(y-y_{n}\right)}^{2}⩽{\mathrm{IR}}^{2}\right\}$$

**Definition 2: Nodeset(C)**. The function to get the set of nodes that deployed in the region *C. Nodeset(C) = {N|(x_n_,y_n_)*∈ *C}*.

**Definition 3: Conflict**. One node could not send and receive packets at the same time; one node could not receive two or more packets at the same time. Any sending and receiving that violates the two principles is called a conflict.

**Definition 4: Conflict neighbor and conflict target**. Essentially, conflict is the reflection of network physical topology and logical topology. For a sender node *N*, a conflict about *N* will occur if and only if: for a node *M* (*M* ≠ *N*), there is a node *P* meeting the conditions that, *P* is in one node’s (*M* or *N*) communication range and another nodes’ interference range; moreover, link *N −> P* and *M −> P* are exist at least one in logical topology. In other words, if *P* should receive one node’s packet, *P* may be interfered by another node’s signal, then, a collision will occur.

For a node *N*, all nodes in its interference range are likely to be conflict locales due to *N*’s transmission action. Then, the definitions of **conflict neighbor** and **conflict target** are given as:

For node *N*, *M* and *P*, if they meet the two conditions at the same time, then *P* is the conflict target of *N* and *M* is the conflict neighbor of *N*.

1. Physical topology condition:
  P∈Nodeset(Cov(N,TR)∩Cov(M,IR))‖P∈Nodeset(Cov(N,IR)∩Cov(M,TR))
2. Logical topology condition:
  (N–>P)‖(M–>P)

According to this definition, all conflict neighbors and conflict targets of a node *N* are in *Cov*(*N*, *TR* + *IR*).

**Figure 2.**
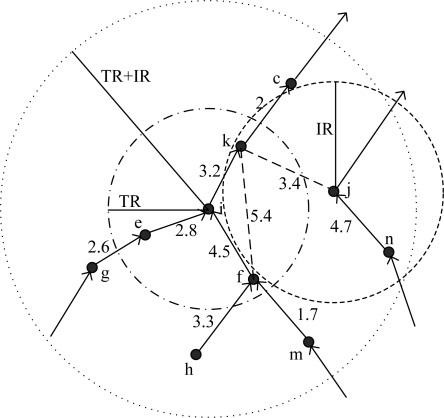
Local network topology of node *i*.

See Figure 2 as an example, *k* is in the intersection of *i*'s transmission range and *j*'s interference range, and link *I* −> *k* is exist, so *j* is *i*'s conflict neighbor and *k* is the corresponding conflict target. However, *n* is far from *i*, and there is no node in the intersection of their communication range and interference range, so they fall short of the physical topology condition, then *n* is not *i*'s conflict neighbor.

**Definition 5: Local conflict graph (LCG).** Node *i*'s local conflict graph *LCG(i)* is a weighted multi-graph with a star like topology, which is used to describe all conflict cases associated with node *i*. Vertex of *LCG(i)* includes node *i* and all its conflict neighbors.

If *j* is *i*'s conflict neighbor and *k* is their conflict target, the weight of link *i* −> *j* in *LCG(i)* is *W_ij_(k)*. Define the propagation delay of link *i* −> *k* and *j* −> k is *D_ik_* and *D_jk_*:

(1) $$W_{\mathrm{ij}}(k)=D_{\mathrm{ik}}-D_{\mathrm{jk}}$$

For standardization, define *D_ii_* = 0. The link weight in LCG could describe a node’s collision state exactly. If *W_ij_(k)* is a positive value, it predicates that, if node *i* chooses the transmission moment *W_ij_(k)* time earlier than *j*, then *i* and *j* will have a conflict at node *k*. Contrarily, negative value of *W_ij_(k)* means that, if node *i* chooses the transmission moment |*W_ij_(k)*| time later than *j*, then *i* and *j* will have a conflict at node *k*.

E.g., in Figure 2, *i* and *j* will collide at *k*, then *j* is a conflict neighbor of *i*. There is a wedge *i−j* in *LCG(i)* and *W_ij_(k)* = 3.2 − 3.4 = −0.2 s. Then if *i* sends packet 0.2 s later than *j*, signals of *i* and *j* will make a conflict at *k*.

For node *i* and *k*, *Nodeset*(*Cov*(*i, IR*) ∩ *Cov*(*k, IR*)) = {*i*, *k*}, however, only the link *i −> k* exist. According to definition 4, k is the conflict neighbor of *i*, and *k* is the conflict target of *i−k*. *W_ik_(k)* = *W_ik_ − W_kk_* = 3.2. If *i* sends packet 3.2 s earlier than *k*, then when *k* begins to send it could not receive the packet from *i*, a collision occurs at *k*.

For node *i* and *f*, *Nodeset*(*Cov*(*i, R_T_*) ∩ *Cov*(*f, R_I_*)) *=* {*i, k, f*}, however, link *i −> f* does not exist, according to definition 4, *f* is not a conflict target. In Figure 2, *f* doesn’t need to receive packets of *i*, so f could send packet when *i*’s packet is arriving at *f*, as a conclusion, *f* is not the conflict target of *i−f* collision. Link *i −> k* exists, so *k* is the conflict target of *i−f* collision. Link *f −> i* exists, then node *i* is also the conflict target of *i−f* collision, because node *i* could not send packets when it is receiving packets of *f*, otherwise a collision will occur. In conclusion, there are two edges connecting *i* and *f* in *LCG(i)*, there weights are: *W_if_(i)* = *D_ii_* − *D_if_*= −4.5 and *W_if_(k)* = *D_ik_−D_fk_* = −2.2.

**Definition 6: forbidden time**. Forbidden time is a time range on the time axis. A node cannot choose a transmission moment in this time range.

**Figure 3.**
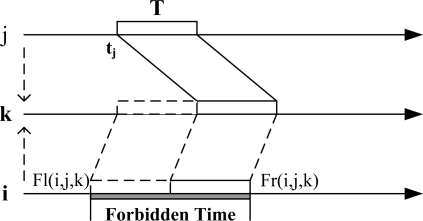
Calculation of forbidden time.

The forbidden time is used to guarantee that the arriving time ranges of two nodes’ frames on the receiver’s time axle do not overlap completely. As shown in Figure 3. In *LCG(i)*, if its neighbor *j* choose the moment *t_j_* as its transmission moment, then *i*’s forbidden time caused by *j* is (*Fl(i, j, k), Fr(i, j, k)*), where *k* is the node that the collision of *i* and *j* may occur:

(2) $$\mathrm{Fl}\left(i,j,k\right)=t_{j}-W_{\mathrm{ij}}(k)-T$$

(3) $$\mathrm{Fr}\left(i,j,k\right)=t_{j}-W_{\mathrm{ij}}(k)+T$$

The physical meaning of forbidden time is that, if a conflict neighbor *j* chooses its transmission moment *t_j_*, node *i* could not choose its transmission moment in (*Fl(i, j, k), Fr(i, j, k)*). See Figure 2 as an example. We assume the length of frame transmission T = 1. For node *i* and *j*, *W_ij_(k)* = −0.2, if *t_j_* = 2, then the forbidden time of *i* caused by *j* is (1.2, 3.2) according to Formula (2) and 3. For node *i* and *f*, assume *t_f_* = 0. Due to the analysis above, there are two weights of edge *i−f* in *LCG(i)*, then the forbidden time of node *i* caused by *f* is (3.5, 5.5)∪(1.2, 3.2), also two periods of time accordingly.

### 3.3. ECS Process

ECS uses two steps to get nodes’ transmission moments. First, each node generates its local conflict graph (LCG). Then each node runs the marking algorithm to calculate transmitting moment on its LCG. The allocation order of transmitting moment depends on node’s marking priority, which is calculated by a group of heuristic rules.

#### 3.3.1. Generating LCG

Each node *i* collects geographical positions of all nodes in *Cov(i, TR*+*IR)* and calculates its local network topology. Then Function 1 is run to generate its local conflict graph according to the rules given by Definition 5.

Definition 4 is used to judge whether a node *j* is a conflict neighbor of *i*. Function PTC() and LTC() are used to judge whether nodes are suit to the physical and logical topology conditions. For each node *j*, if there exists a node *k* satisfies that, *j* is the conflict neighbor of *i* and *k* is their conflict target, then *W_ij_(k)* = *Dik* − *Djk* is calculated and added in *LCG(i)*.

Let *S_LCG_(i)*=*{(i, j, k, W_ij_(k))*| *i, j, k∈Cov(i, TR*+*IR)}* and *V(i)*=*{m*|*m∈Cov(i, TR*+*IR)*.

**Figure 4.**
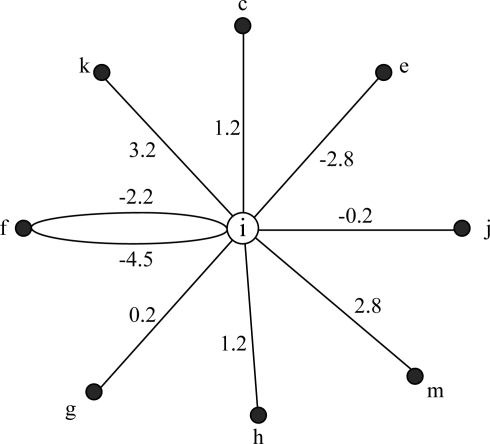
Local conflict graph (LCG) of node *i*.

In the case of Figure 2, *LCG(i)* is shown in Figure 4. According to the analysis above, node *i* and *f* have multi edges in Figure 4. Due to the multiplicity of forbidden time, LCG is a multi-graph. Namely, if two nodes may make collisions at more than one conflict targets, they will have multi edges in LCG. The number of multi edges is equal to the number of conflict targets.

**Function 1.** CreateLCG().

1: Input: *V(i)*
2: Output: *S_LCG_(i)*
3: **PTC**(*N, P, M*){ //physical topology condition judgment
4: if (*P*∈*Nodeset*(*Cov*(*N, R_T_*)∩*Cov*(*M, R_I_*)) ‖ *P*∈*Nodeset*(*Cov*(*N, R_I_*)∩*Cov*(*M, R_T_*)))
5: return *true*
6: else
7: return *false*
8: }
9: **LTC**(*N, P, M*){ //logical topology condition judgment
10: if(∃*N*->*P* ‖∃*M*->*P*)
11: return *true*
12: else
13: return *false*
14: }
**createLCG(i)**
15: new *V_LCG_*= *V(i)*;
16: while (*V_LCG_* != *null*) {
17: ∀*j*∈*V(i)* //Create a new edge *i*->*j*
18: *V_LCG_=V_LCG_-{j};*
19: new *V’=V(i);* // Traverse all neighbors to create all multi-edges related to *i* and *j*
20: while (*V’* != *null*) {
21 ∀*k*∈*V’*
22: *V’=V’-{k}*;
23: If (*PTC(i, k, j)=true && LTC(i, k, j)=true*) {
24: *W_ij_(k)=D_ik_−D_jk_*;
25: *S_LCG_(i)=S_LCG_(i)∪{(i, j, k, W_ij_(k))};*
26: } //end if
27: } //end while (*V’* != *null*)
28: } //end while(*V_LCG_* != *null*)
29: return *S_LCG_(i)*;

#### 3.3.2. Choosing Transmission Moment

After generating the local conflict graph, each node calculates its marking priority, and then begins to choose its transmission moment. The node with the highest priority will mark its transmission moment firstly; the basic idea is to choose the earliest available time on its time axle (keep away from forbidden time). The main process of ECS contains five steps:

Step 1: Each node generates its LCG and calculates its marking priority. Each node run step 2–5 in distributed manner.
Step 2: Node broadcasts its priority to all LCG neighbors. All the nodes set the status to ‘unmarked’.
Step 3: If the node has the highest marking priority in all its unmarked LCG neighbors, it calculates its transmitting moment and broadcasts it to all LCG neighbors, and then sets the status to ‘marked’.
Step 4: If exists a higher priority neighbor for a node, it must wait for its neighbor to mark and receive the neighbor’s transmission moment, then update its forbidden time due to the neighbor’s selection. Return to step 3.
Step 5: If all nodes in LCG are ‘marked’, the algorithm stops.

Define *S_c_(i)* as the set of all marked conflict neighbors of node *i* and *S_uc_(i)* is the set of all unmarked conflict neighbors; *ST(i)* is the status of node *i* (*marked* or *unmarked*) and *PRI(i)* is the marking priority of node *i*. Function 2 is used to choose transmission moments (step 2–5).

**Function 2.** Mark().

**Initialization:**
1: *S_c_(i)=null*, *ST(i)=unmarked*;
2: *S_uc_(i)*={*j*|*j* is *i*’s LCG neighbor}
**Mark(i)**
3: while(∃*j*∈*S_uc_(i)*, *PRI(i)<PRI(j)*){ //Not highest priority, wait and receive
4: receve node *j*’s selection *t_j_*
5: *S_c_(i)=S_c_(i)∪{j}*
6: *S_uc_(i)=S_uc_(i)-{j}*
7: for each *j*
8: calculate *Fl(i, j, k)* and *Fr(i, j, k)* //Forbidden time
9: } // end while
10: loop: //highest priority; ordinal marking, find the earliest available time
11: *t_i_ =*0;
12: for each *j∈S_c_(i)*{
13: if(*Fl(i, j, k)<t_i_<Fr(i, j, k)*)
14: *t_i_ = Fr(i, j, k)*;
15: }
16: }
17: *ST (i)* = *marked*;
18: return *t_i_*;

The length of a TDMA period is decided by *max(t_i_* + *D_ik_)*, where *k* is the next-hop node on the path to base station of *i*. This value should be broadcasted to the whole network. For each node *i*, define *T_p_(i)* = *t_i_* + *D_ik_*. After choosing transmission moment, each node begins to broadcast its *T_p_* to neighbors. If a node receives a larger *T_p_*, it begins to broadcast this value and abandons smaller ones. Define a period of time *T_wait_*, if a node has not receive a larger *T_p_* for more than *T_wait_*, it will regard its current *T_p_* as the length of a TDMA period. Therefore, *T_wait_* must be long enough and is decided by the network scale.

#### 3.3.3. Heuristic Rules

In UWSN environments, allocating continuous time to all nodes is equivalent to the problem of distribute coloring on a continuous color axis, which is a well known NP-hard problem to ascertain the optimum global allocating scheme [23]. The goal of ECS is to improve channel utilization and transmission parallelism as far as possible. As marking priority is used, the heuristic rules to calculate priority will affect algorithm efficiency directly. Therefore, in ECS three heuristic rules are proposed to calculate nodes’ marking priority. The importance of three rules reduces in turn.

1. Nodes that have largest degree in its LCG should be marked preferentially. A node’s degree is the number of edges in its LCG. A node that has large degree predicates that it could collide with large amount of nodes. Therefore, this kind of node should transmit as early as possible to move up other nodes’ forbidden time. Then the length of transmission period of the network can be shortened.
2. Nodes that have higher traffic load should transmit as early as possible. A node has high traffic load will take more time to transmit, so it should also transmit earlier to reduce average packet delay.
3. Nodes with larger propagation delay link should transmit earlier. If the large latency link transmits late, the receiver will get the packet even later. It is helpful to improve transmission parallelism if large latency link transmits earlier and small latency link transmits later.

Marking priority could be calculated due to the three heuristic rules. Define *PRI(i)* as node *i*’s marking priority, *d_i_* is node *i*’s degree in *LCG(i)*, *L_max_* is the maximum queue length for a node, and *L_i_* is node *i*’s traffic load (not exceeding *L_max_*). Node *k* is *i*’s next-hop node on the path to base station. *D_ik_* is the propagation delay of link *i − k* and *D_max_* is the maximum propagation delay in the network. The propagation delay of a link is the ratio of link distance and the speed of submarine sound signal:

(4) $$\mathrm{PRI}(i)=d_{i}\times L_{\text{max}}+L_{i}+\frac{D_{\mathrm{ik}}}{D_{\text{max}}}$$

The importance of different parameters is reflected in Formula (4). The node with largest degree will always have the highest priority. If the degree is equal, the one with heavier load will be marked preferentially, and if the load is also equal, the one with larger propagation delay will have higher priority.

### 3.4. An Instance for ECS

**Figure 5.**
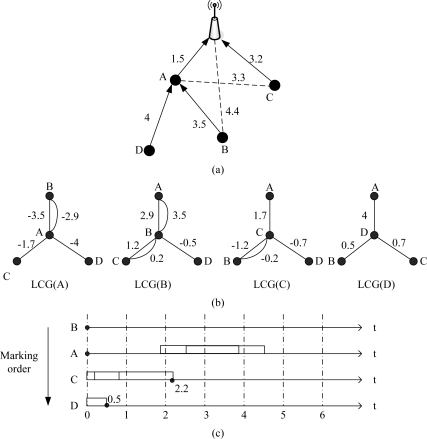
ECS process: **(a)** network topology; **(b)** nodes’ LCG; **(c)** marking order and results.

In this subsection, we give an example to illustrate the process of ECS in detail. In Figure 5(a), frame time is 1 second, and propagation delay of each link is marked in the figure. First, each node runs Function 1 and generates its local conflict graph, shown in Figure 5(b).

Second, all nodes calculate their marking priority. In Figure 5(b) *B* has the largest degree; *A* and *C* have same degree, however, assume the rate of generating packets for each node is equal, the traffic load of *A* is higher than *C* (because *A* has two child nodes and should relay their packets), then priority of *A* is higher than *C*; *D* has the smallest degree. As a result, the final marking order is *B*, *A*, *C* and *D*.

*B* chooses 0 as its transmission moment according to the marking rules and broadcasts to *A*, *C* and *D*, then *A* calculates its forbidden time caused by *B*: (1.9, 3.9)∪ (2.5, 4.5), so *A* also chooses 0; *C* calculates its forbidden time due to the transmission moments of *A* and *B*, the result is (−2.7, −0.7)∪ (0.2, 2.2)∪ (−0.8, 1.2), then *C* chooses 2.2; *D* calculates its forbidden time caused by *A*, *B* and *C*: (−5, −3)∪ (−1.5, 0.5), then *D* chooses 0.5. The forbidden time is shown in Figure 5(c). Finally, *A*, *B*, *C* and *D* set their transmission moments as 0, 0, 2.2, 0.5.

As a conclusion of this instance, ECS could improve network transmission parallelism. Both *A* and *B* send packets at time 0 but do not make a conflict, which is impossible in TSN. In UWSNs, the propagation delay is long and packet length is short, then the arriving time of packets from different nodes is disparate. ECS uses this special phenomenon and improves network throughput.

### 3.5. Maintenance

UWSNs are deployed in severe environments. In the harsh underwater environment links will be intermittent and nodes may be mangled or be divorced from the network because of many uncertainties. The security and reliability of UWSNs is worse than that of TSNs, so deploying new nodes and changing network topology are frequent in UWSNs. As nodes’ disappearance and entering are usual, network protocols must be designed flexibly and vigorous enough to suit to these changes. However, compared with competition based protocols, the adaptability of scheduling based protocols is worse, because strategy is usually pre-established and could not change after network beginning, then existing TDMA protocols are almost not involving network maintenance, including ST-MAC.

ECS uses a flexible and simple scheme to deal with the change of network topology. Nodes’ death or entering denotes the reallocation of transmission moment. The conversion of a node’s transmission moment will cause ripple effect in the whole network. Therefore, the key problem is to keep the effect of topology change in local scope.

**Figure 6.**
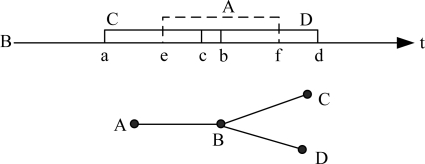
A node may be incapable to use the transmission time of a dead node.

If a node does not receive packets of its child node in continuous *m* rounds, it considers the child node is dead. Then it broadcasts this message to all the child’s LCG neighbors. Other nodes could utilize the transmission time of dead node. ECS makes the node with highest priority to use the transmission time of dead node. However, due to the complexity of UWSN network topology and great divergence of link delay, a node may not use this time, as shown in Figure 6.

In the example of Figure 6, the forbidden time of *A*, *C*, *D* to *B* is (e, f), (a, b) and (c, d). If *A* is dead and *B* is the highest load node in *A*’s LCG, *B* should use *A*’s transmission time and choose a new transmission moment in (e, f). However, this time is still forbidden by node *C* and *D*, and then *B* cannot utilize it. If a node could not use the time of dead node, the one whose priority is second to it will request this time, and so on.

Function 3 is the pseudo-code to demonstrate how to deal with an exhausted node. For nodes that are not in communication range, messages should be delivered by multi-hop routing.

**Function 3.** Maintenance().

1: If a node *j* don’t receive packet of its child node *i* for continuous *m* rounds
2: Broadcast message ‘i_death’ to *V(i)*;
3: Loop{
4: choose the highest priority node *k* in *V(i)*;
5: if(∃*t_k_*∈*F(k, i, x)* and ∃*a*, *b*∈*V(k)*, *Fl(k, a, y)*<*t_k_*<*Fr(k, a, y)*&&*Fl(k, b, z)*<*t_k_*<*Fr(k, b, z)*)
6: choose the node that priority is second to *k // k* couldn’t use the transmission time of *i*
7: continue loop;
8: else
9: calculate *t_k_*; // Choose the earliest available moment in *F(k, i, x)*
10: break;
11: }
12: broadcast *t_k_* to *V(k)*;

If an old node rejoins in the network, e.g., link quality is improved or the node recovers from faults. Nodes that occupy its transmission moment should abdicate and the network returns to previous scheduling. If a new node joins in the network, it must keep jamming the channel for one TDMA period to inform its neighbors. Then in next period, the neighbors send their transmission moments and geographical positions to the new node. According to the regulations of ECS, the new node must know all information in its interference range to generate its local topology. New node calculates all of its forbidden time, and chooses the earliest available time as its transmission moment, then broadcasts to all LCG neighbors. If the new node could not find transmission moment on time axle, it turns to sleep and becomes a spare node. This case is unfamiliar because new nodes are usually deployed in sparse region or the region that has dead nodes. Function 4 is the pseudo-code of a new node to get its transmission moment.

**Function 4.** Join(i).

1: jam the channel;
2: receive information of all LCG neighbors and calculate their forbidden time, create LCG(i);
3: *t_i_*=0;
4: for (all node *j* in *LCG(i)*) {
5: if(∃*k*∈*LCG(i)*, *Fl(i, j, k)<t_i_<Fr(i, j, k)*)
6: *t_i_= Fr(i, j, k)*
7: }
8: if (*t_i_*>*t_max_*)
9: node *i* turns to sleep, break; // Spare node
10: else{
11: broadcast *t_i_* to all LCG neighbors;
12: return *t_i_*;
13: }

## 4. Simulation

In this section, we describe simulation conditions and results, and analyze the performance of ECS in terms of topology and throughput. Emulator is run in Matlab software. In our simulation the frame time *T* is set to 1 s; nodes’ communication range is 3,000 m and interference range is 3,500 m. Network scale changes from 5 to 60 nodes. Nodes are deployed in the area no more than 12 km × 12 km, and link distance is restricted from 450 m to 3,000 m by setting different deployment density. As the speed of submarine sound signal is 1,500 m/s, the propagation delays of links are form 0.3 s to 2 s.

Network throughput is defined as the number of packets transmitted in the network per second. For network topology has great relationship with transmission parallelism, we simulated both common topology (nodes are deployed randomly and self-organized to aggregation tree) and special topology such as line, star and grid.

Four communication-scheduling algorithms are simulated in the simulations:

1. Optimal: the theoretically most excellent scheme, it chooses the scheme with the shortest TDMA super frame among all possible schemes, which means nodes use the shortest time to achieve transmission in a round. Therefore, it has the highest throughput and gives the upper bound of all heuristic algorithms.
2. ECS: the continuous time allocation based TDMA protocol proposed in this paper.
3. ST-MAC: multi-slot TDMA protocol proposed in [8].
4. S-TDMA: a classical traditional single slot TDMA [16]. The length of a time slot is set to frame time plus to longest propagation delay in the network.

**Figure 7.**
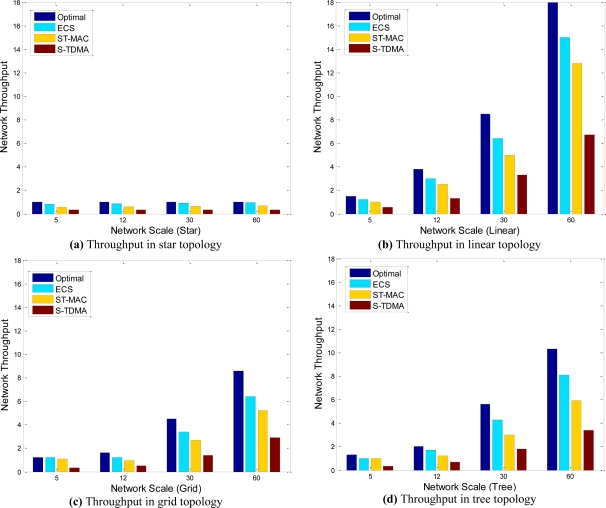
Network throughput in different topologies: **(a)** Star, **(b)** Linear, **(c)** Grid, **(d)** Tree.

Figure 7 illustrates network throughput of the four algorithms in four different topologies. They show that network topology has the great relationship with network throughput. For linear, tree, grid and star topology, throughput decreases in turn. Because as the change of topology, node’s density increases gradually, more and more nodes share the same channel, and then less and less nodes could transmit concurrently.

Network throughput increases as network scale increases in any topology except star. The reason of this special phenomenon is that, in star topology all nodes transmit to the sink by one hop, any two nodes are potential conflict nodes. In other words, all nodes only share one channel, so transmission parallelism cannot be improved by network scale.

When the network scale is small, ECS and ST-MAC both perform close to Optimal. However, as the network scale becomes large, both of them perform worse than Optimal, but ECS is always better than ST-MAC (20% better on average), and achieves 80% capability of Optimal. S-TDMA is the worst strategy. Large idle time exist in S-TDMA because of its long time slot.

ST-MAC uses a slot scheme to allocate transmission moments for all nodes. Any packet must be sent at the beginning of a slot, so it is a conservative strategy compared with continuous time allocation. Moreover, ST-MAC works under the assumption that the propagation delay of any link must be an integral multiple of the frame time, so the frame size must be designed as small as possible. Short data packets will decrease the scale of valid data, because other information such as parity bit and frame header is not become shorter when data packet becomes short. ECS uses continuous time allocation scheme and do not need the assumption of ST-MAC, so ECS could use longer data packet and further improve network throughput.

**Figure 8.**
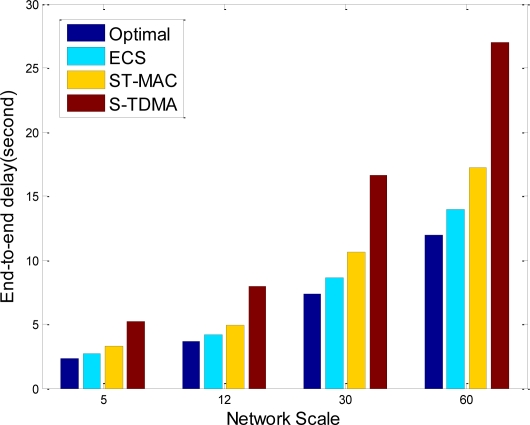
End-to-end delay of different TDMA protocols.

End to end delay is also considered in simulations, and the results in popular tree topology are shown in Figure 8. In sensor networks data is collected by base station, so end-to-end delay is the latency of data propagation from source node to base station. In Figure 8, end-to-end delay increases as network scale increases, because node to sink average hops are increasing. ECS uses continuous time allocation scheme and reduces idle time of receiver nodes, so its end-to-end delay has reduced 18%, and only 12% larger than optimal scheme. Compared with traditional TDMA protocols, ECS has higher network throughput and lower end-to-end delay.

ST-MAC and ECS are both TDMA based protocols and have no collision in data transmission phase. Therefore, their energy efficiency is both high. The additional energy consumption is only come from initialization phase. In ST-MAC, base station should collect information and dispense control message to the whole network. Any node should communicate with the base station by multi-hop transmission. The whole communication cost is O(N!) level. However, ECS is a distribute algorithm, node could only exchange information with local neighbors, and the whole communication cost is O(N) level, where N is the network scale. When network scale becomes large, the communication cost of ST-MAC is pretty much more than that of ECS.

As a conclusion, ECS has achieved a good tradeoff for network throughput and communication cost for protocol running. The performance of distribute and heuristic allocation algorithm is close to theoretically optimal scheme. What’s more, it performs better than existing approaches such as ST-MAC.

**Figure 9.**
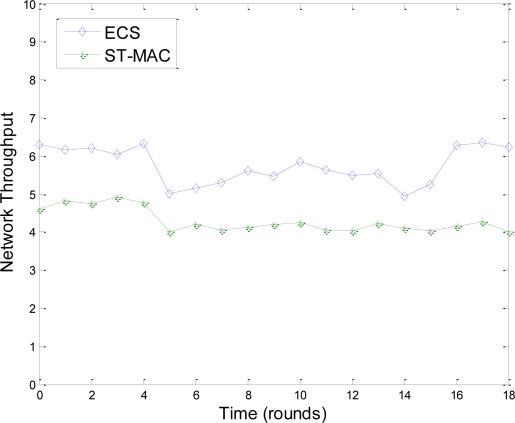
Efficiency of ECS in underwater harsh environments.

Figure 9 shows a comparison of ECS and ST-MAC in harsh environments. The network has 60 nodes and tree topology. We use lossy link and the transmission probability is set to 80%. In simulation, we randomly let 10 nodes temporarily drop out of network for about three minutes. Figure 9 shows that, in ECS, during the period of nodes’ disappearance, their neighbors utilize transmission moments of these nodes and throughput is improved. Throughput resumes quickly when the 10 nodes rejoin the network. However, the throughput of ST-MAC without a maintenance scheme could not resume. As a conclusion, in underwater harsh environments, topology change has little effect on ECS because of the maintenance scheme.

## 5. Conclusions

In this paper, we have proposed an efficient TDMA protocol (ECS) for UWSNs, including the continuous time based and sender oriented conflict analysis model, the transmission moment allocation algorithm and the distributed topology maintenance algorithm.

ECS is different from previous TDMA approaches in allocating transmission moments to nodes based on continuous time, not on discrete time slots. ECS exploits well the characteristics of acoustic signal propagation such as long propagation delay and long communication rage. By using continuous time based transmission moment allocation scheme, differences of link delays are further utilized and channel utilization of receiver node is improved. Simulation results confirm that compared with traditional slotted TDMA protocols, ECS has higher network throughput and better efficiency.
